# Synchronous multiple primary colorectal cancer with discordant RAS status between primary lesions: a case report in an elderly patient

**DOI:** 10.3389/fsurg.2026.1884577

**Published:** 2026-08-17

**Authors:** Junting Guan, Jiyuan Chen, Shengqi Pan, Qi Li, Guiyu Wang, Qingchao Tang

**Affiliations:** Department of Colorectal Surgery, The Second Affiliated Hospital of Harbin Medical University, Harbin, Heilongjiang, China

**Keywords:** case report, colorectal neoplasms, elderly patient, intratumoral heterogeneity, microwave ablation, multiple primary neoplasms, ras genes

## Abstract

**Background:**

Synchronous multiple primary colorectal cancer (SMPCRC) is rare and often shows molecular heterogeneity between primary lesions. Treatment decisions are particularly challenging in complex scenarios involving multiple independent lesions, distant metastasis, and advanced age with intolerance to standard chemotherapy.

**Case presentation:**

A 77-year-old man presented with hematochezia and was diagnosed with four independent neoplastic lesions within three months: a primary rectal lesion (pT3N1aM0), a sigmoid colon primary carcinoma, a proximal rectal adenoma with focal intramucosal carcinoma, and an adenocarcinoma of the transverse colon near the splenic flexure [resected by endoscopic submucosal dissection (ESD) three months later]. Bilateral hepatic metastases were detected six months postoperatively, with the carcinoembryonic antigen (CEA) level still within the reference range at diagnosis. Molecular testing of the two primary carcinomas revealed marked discordance: the primary rectal lesion carried mutation signals in both KRAS exon 4 and NRAS exon 2, whereas the sigmoid colon lesion was entirely RAS/RAF/PIK3CA wild-type — testing either lesion alone would have led to opposite anti-EGFR treatment decisions. Given the patient's advanced age and intolerance to standard chemotherapy, a staged, multimodal integrated strategy was adopted: synchronous surgery for three primary lesions, minimally invasive ESD of the transverse colon lesion, artificial ascites-assisted percutaneous microwave ablation (AA-MWA) for liver metastases, and individualized adjustment of chemotherapy (switched from CAPEOX to oxaliplatin plus raltitrexed because of intolerance). Over 18 months of follow-up, no radiological recurrence was observed and the patient remained in good general condition.

**Conclusions:**

This case demonstrates that primary lesions in SMPCRC can harbor substantial molecular heterogeneity, such that single-lesion testing may be insufficient to guide treatment decisions in complex scenarios. For elderly patients intolerant to standard regimens, a staged, multimodal integrated approach (synchronous surgery + ESD + AA-MWA + individualized chemotherapy adjustment) may serve as a feasible comprehensive treatment option.

## 1 Introduction

Colorectal cancer (CRC) is the third most common malignancy worldwide and the second leading cause of cancer-related death (1). Synchronous multiple primary colorectal cancer (SMPCRC) is a special subtype, defined as the simultaneous presence (within an interval of less than six months) of two or more independent primary CRC lesions in the same patient (2). SMPCRC accounts for approximately 1%–8% of all CRC cases (3), and three or more synchronous primary lesions are even rarer. The development of multiple primary CRC is associated with hereditary tumor syndromes (such as Lynch syndrome and familial adenomatous polyposis), chronic inflammatory bowel disease, and advanced age; however, a substantial proportion of cases are sporadic, and the underlying molecular mechanisms remain incompletely understood (4).

In the precision treatment of metastatic CRC, the mutation status of the RAS genes (KRAS and NRAS) is a key molecular marker determining the indication for anti-epidermal growth factor receptor (EGFR) monoclonal antibodies (cetuximab, panitumumab) (5). According to the current National Comprehensive Cancer Network (NCCN) guidelines, patients carrying any KRAS (exon 2, 3, 4) or NRAS (exon 2, 3, 4) mutation should not receive anti-EGFR therapy (6). However, in patients with SMPCRC, significant genetic heterogeneity may exist between multiple primary lesions. In a study of eight patients with SMPCRC, de Macedo et al. (7) found that 75% of cases showed discordant RAS mutation status between different primary lesions, suggesting that testing only a single lesion may lead to incorrect treatment decisions. Nevertheless, current mainstream guidelines (such as NCCN and ESMO) do not provide explicit recommendations for multi-lesion molecular testing in SMPCRC (5, 6), and some reports still base treatment decisions on the molecular features of the index tumor (8), suggesting that the strategy of testing multiple lesions separately may not yet be widely adopted in current clinical practice.

Non-canonical variants in KRAS exon 4 (codons 117/146) and NRAS exon 2 (codons 12/13) both occur at low frequencies in CRC (9, 10). A study of 1,110 Chinese CRC patients showed that the overall mutation rate of KRAS exons 2/3/4 was approximately 45.4%, with exon 4 (mainly A146 and K117) accounting for only a minority; the overall NRAS mutation rate was approximately 3.9%, with exon 2 accounting for a portion of NRAS mutations (10). Concurrent KRAS and NRAS mutations are uncommon in CRC — Zelli et al. (11) found only one such case (0.6%) among 161 CRC patients. In SMPCRC, a pattern in which one primary lesion carries mutation signals in both KRAS exon 4 and NRAS exon 2 while another primary lesion is entirely wild-type has, to our knowledge, rarely been documented; the value of the present report lies less in the specific combination of variants than in the degree of inter-lesional heterogeneity it reveals and its direct implications for treatment decision-making.

In addition, the early detection of CRC liver metastasis still relies mainly on dynamic monitoring of carcinoembryonic antigen (CEA) and periodic radiological evaluation; however, previous studies have shown that CEA has limited sensitivity for detecting CRC recurrence, with a reported sensitivity of only approximately 50%–80%, meaning that a substantial proportion of recurrences or metastases may occur without an elevation in CEA (12, 13), highlighting the importance of radiological surveillance in CEA-negative populations.

Here we report a 77-year-old man with SMPCRC presenting with four independent neoplastic lesions (a primary rectal lesion, a sigmoid colon carcinoma, a transverse colon adenocarcinoma, and a proximal rectal adenoma with focal carcinoma), with marked discordance in RAS status between primary lesions — the primary rectal lesion carried mutation signals in both KRAS exon 4 and NRAS exon 2, whereas the sigmoid colon lesion was entirely RAS/RAF/PIK3CA wild-type; liver metastasis was detected by radiological evaluation while the CEA level was still within the reference range. Through an integrated strategy of synchronous surgery, ESD, AA-MWA, and individualized chemotherapy adjustment, the patient achieved stable disease control for more than 18 months. The significance of this report is twofold: (1) it illustrates the degree of complexity that molecular heterogeneity between primary lesions in SMPCRC can reach, and the potential for single-lesion testing to mislead treatment decisions; and (2) in the complex clinical scenario of “advanced age + multiple lesions + secondary liver metastasis + intolerance to standard chemotherapy,” it provides a generalizable model of multimodal integrated treatment.

## 2 Case presentation

### 2.1 General information

A 77-year-old man was admitted in September 2024 because of “intermittent blood in the stool for 2 months.” He had a more than 10-year history of hypertension, well controlled with regular medication, and had undergone appendectomy for acute appendicitis 30 years earlier. He had no history of smoking or alcohol use and denied any family history of colorectal cancer or other malignancies. On admission, his general condition was good, with an Eastern Cooperative Oncology Group (ECOG) performance status score of 1.

### 2.2 First admission, examination, and surgery: synchronous management of multiple colorectal lesions

After admission, colonoscopy and contrast-enhanced upper abdominal magnetic resonance imaging (MRI) were completed; the colonoscope could not pass beyond the segment proximal to the obstructing rectal tumor. The patient underwent surgery. Intraoperative exploration revealed a tumor of approximately 3 cm × 3 cm × 4 cm in the sigmoid colon (not detected on preoperative colonoscopy because the colonoscope could not pass the obstructing rectal tumor), along with extensive multiple small polyps within the bowel. Multiple small polyps were distributed across several colorectal segments (cecum, ascending, transverse and descending colon, sigmoid colon, and rectum); on subsequent surveillance colonoscopies the polyps recurred and were removed endoscopically, with up to 21 polyps detected at a single examination and several dozen removed in total over repeated follow-up, ranging mostly from 0.3 to 1.5 cm in size. After comprehensive assessment, the following procedures were performed: (1) transanal resection of an adenomatous lesion 3 cm from the anal verge (intraoperative frozen section indicated adenoma); (2) laparoscopic radical resection of rectal cancer (for the primary lesion 8 cm from the anal verge); (3) laparoscopic radical resection of sigmoid colon cancer; and (4) loop ileostomy.

**Figure 1:**
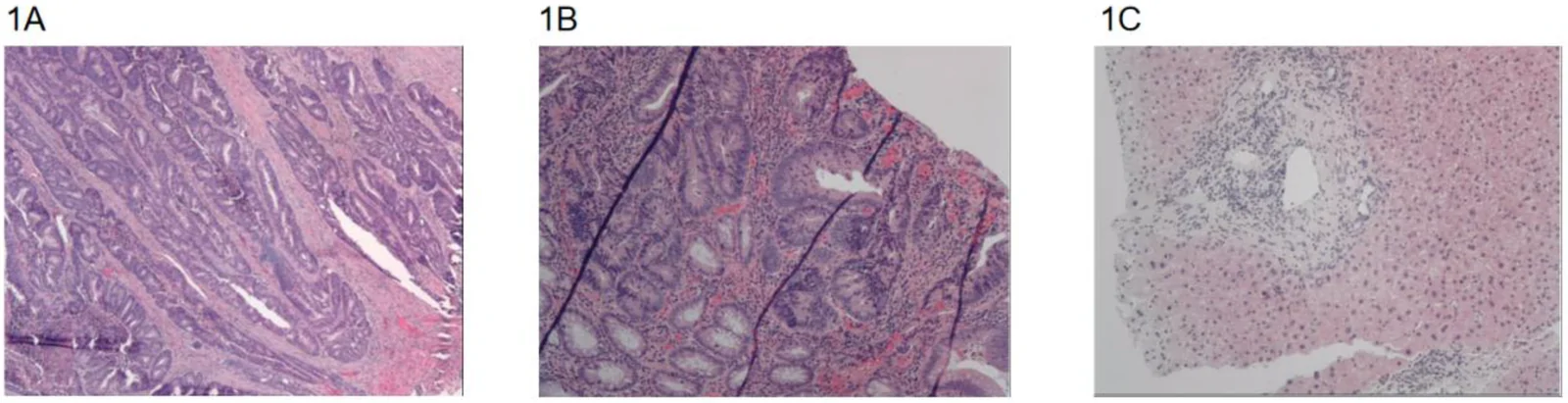
Histopathology of the tumor lesions (HE staining). **(A)** Primary rectal lesion (8 cm from the anal verge), moderately differentiated adenocarcinoma with infiltrative glandular structures. **(B)** ESD specimen of the transverse colon lesion near the splenic flexure, adenocarcinoma with a positive basal margin. **(C)** Liver metastasis biopsy, scattered adenocarcinoma infiltrating the hepatic parenchyma with inflammatory cell infiltration.

Postoperative pathology: the primary rectal lesion was a protruding moderately differentiated adenocarcinoma (pT3N1aM0, 1/14 pericolonic lymph nodes positive, Figure 1A); the sigmoid colon lesion was a protruding moderately differentiated adenocarcinoma invading the subserosa; the lesion 3 cm from the anal verge was a tubular adenoma with focal intramucosal carcinoma, with a negative basal margin, the carcinomatous component confined to the mucosa without submucosal invasion, and transanal local resection achieving oncologically complete excision; multiple hyperplastic polyps were seen in the rectum and sigmoid colon. On histological examination the great majority of the resected polyps were hyperplastic, with only a small number of tubular adenomas showing varying degrees of dysplasia, and the overall appearance did not amount to a carpet of adenomas typical of familial adenomatous polyposis. Mismatch repair (MMR) immunohistochemistry showed intact expression of MLH1, MSH2, MSH6, and PMS2, indicating pMMR/MSS and essentially excluding Lynch syndrome. The stoma passed gas on postoperative day 1; symptoms of stomal obstruction appeared on day 2 and resolved after acupuncture treatment.

### 2.3 Detection and management of the fourth neoplastic lesion in the transverse colon

Approximately three months after the first operation, the patient was scheduled for ileostomy reversal. Preoperative colonoscopy revealed a tumor of approximately 2 cm in the transverse colon near the splenic flexure, which was resected by endoscopic submucosal dissection (ESD). Pathology reported adenocarcinoma with a positive basal margin (Figure 1B). This lesion was the fourth independent neoplastic lesion in this case (following the rectal cancer 8 cm from the anal verge, the sigmoid colon cancer, and the adenoma with focal intramucosal carcinoma 3 cm from the anal verge), still within the 6-month time window of the Warren-Gates definition of synchronous multiple primary tumors (2). Ileostomy reversal was performed at the same time; the patient passed gas on postoperative day 6 and recovered uneventfully.

Regarding the management strategy for the transverse colon lesion, after the multidisciplinary team thoroughly assessed the patient's advanced age, overall tumor burden, and the risk of repeat laparotomy, and after detailed communication with the patient and family, the decision was made not to perform salvage surgery but to adopt close endoscopic and radiological follow-up. Colonoscopy three months after ESD with biopsy of the original ESD scar reported inflammatory changes without tumor recurrence, indicating effective ESD resection; no evidence of local recurrence was found at this site during subsequent follow-up.

In summary, the four independent neoplastic lesions in this case were detected successively within three months: three were found at the first admission by colonoscopy and intraoperative exploration (the primary rectal lesion 8 cm from the anal verge, the adenoma with focal intramucosal carcinoma 3 cm from the anal verge, and the intraoperatively detected sigmoid colon cancer), and the fourth (transverse colon adenocarcinoma near the splenic flexure) was found on follow-up colonoscopy before the planned ileostomy reversal three months after the first operation; all diagnoses fell within the 6-month time window of the Warren-Gates definition of synchronous multiple primary tumors (2).

### 2.4 Diagnosis of liver metastasis

Approximately six months after the first operation, the patient was admitted because of “abdominal pain for 3 days.” Repeat contrast-enhanced upper abdominal MRI revealed multiple nodular abnormal signals within the liver, the largest located in the right anterior lobe, measuring approximately 14 mm × 10 mm × 15 mm, showing no definite enhancement on any phase of gadoxetic acid-enhanced imaging and no uptake in the hepatobiliary phase, consistent with typical features of metastasis. Notably, the CEA level at this time was only 3.25 ng/mL, within the reference range.

To clarify the nature of the lesions, ultrasound-guided liver biopsy was performed. Pathology showed a small amount of adenocarcinoma (Figure 1C), with immunohistochemistry results of CK20(+), CK8/18(+), CDX2(+), SATB2(+), CK7(-), and Hepatocyte(-), consistent with the typical immunophenotype of CRC liver metastasis; Hepatocyte(-) definitively excluded primary hepatocellular carcinoma. No liver metastasectomy was performed in this patient; because the diagnostic biopsy yielded only a small amount of adenocarcinoma, no residual tissue was available for molecular analysis of the metastatic lesion.

### 2.5 Genetic testing results of the primary lesions

To guide subsequent treatment, RAS/RAF/PIK3CA gene testing was performed separately on formalin-fixed paraffin-embedded (FFPE) tissue from the primary rectal lesion (8 cm from the anal verge) and the sigmoid colon tumor, using a PCR fluorescent probe method. The results are shown in Table 1.

**Table 1:** Genetic testing results of the primary rectal lesion and the sigmoid colon primary tumor.

Test locus	Primary rectal lesion	Sigmoid colon tumor
KRAS exon 2 (codon 12/13)	No mutation	No mutation
KRAS exon 3 (codon 61)	No mutation	No mutation
KRAS exon 4 (codon 117/146)	Mutation signal (K117N/A146T/A146 V/A146P)	No mutation
NRAS exon 2 (codon 12/13)	Mutation signal (G13R/G12C/G12 V/G12A/G13 V/G13C)	No mutation
NRAS exon 3 (codon 61)	No mutation	No mutation
NRAS exon 4 (codon 146)	No mutation	No mutation
PIK3CA exon 20 (H1047R/L)	No mutation	No mutation
BRAF exon 15 (V600)	No mutation	No mutation

Testing was performed using a PCR fluorescent probe method. The NRAS exon 2 probe set simultaneously reports positive signals for multiple loci (G13R/G12C/G12 V/G12A/G13 V/G13C), reflecting the presence of a variant within the region covered by the probe set; this should not be interpreted as concurrent mutation at all six amino acid loci. Identification of the specific mutated locus still requires confirmation by Sanger sequencing or next-generation sequencing (NGS).

The primary rectal lesion showed mutation signals in both the KRAS exon 4 (K117/A146 probe set) and NRAS exon 2 (codon 12/13 probe set) regions, whereas the sigmoid colon tumor was entirely RAS/RAF/PIK3CA wild-type, indicating marked discordance in RAS status between the two primary lesions.

### 2.6 Microwave ablation treatment of liver metastasis

After the diagnosis of liver metastasis, ultrasound-guided microwave ablation of the multiple liver lesions was performed. Preoperative contrast-enhanced ultrasound (CEUS) showed bilobar hypoechoic solid lesions in the liver, with heterogeneous hypoenhancement in the arterial phase and hypoenhancement in the portal and delayed phases, consistent with typical features of CRC liver metastasis.

Considering that the right hepatic lobe lesion was located subcapsularly and posed a relatively high risk to adjacent organs, artificial ascites-assisted percutaneous microwave ablation (AA-MWA) was adopted: normal saline was injected into the peritoneal cavity to separate the liver from the peritoneum and surrounding tissues, after which microwave ablation of the subcapsular lesion in the right hepatic lobe and the lesion in the left hepatic lobe was performed separately under ultrasound and CEUS guidance. The procedure was uneventful, with no obvious complications.

### 2.7 Sequential chemotherapy and regimen adjustment

Approximately one week after ablation, CAPEOX (capecitabine plus oxaliplatin) chemotherapy was started on a biweekly schedule, using a once-every-2-weeks biweekly regimen to accommodate the tolerance of an elderly patient. However, after completing two cycles of treatment, the patient developed severe diarrhea (CTCAE grade 3), indicating poor tolerance to fluoropyrimidines. DPYD genotyping or DPD phenotyping had not been performed before capecitabine initiation.

Considering the patient's advanced age, no prior use of antiangiogenic agents, and intolerance to CAPEOX, combined with the fact that the RAS mutation signals in the primary rectal lesion excluded the indication for anti-EGFR therapy and pMMR/MSS excluded the indication for immunotherapy, the regimen was adjusted after multidisciplinary discussion to a biweekly regimen of oxaliplatin plus raltitrexed — replacing capecitabine with raltitrexed (a thymidylate synthase inhibitor). This regimen was completed for a total of six cycles with good tolerance. Given the patient's advanced age and overall condition, bevacizumab was not added.

### 2.8 Follow-up results

**Figure 2:**
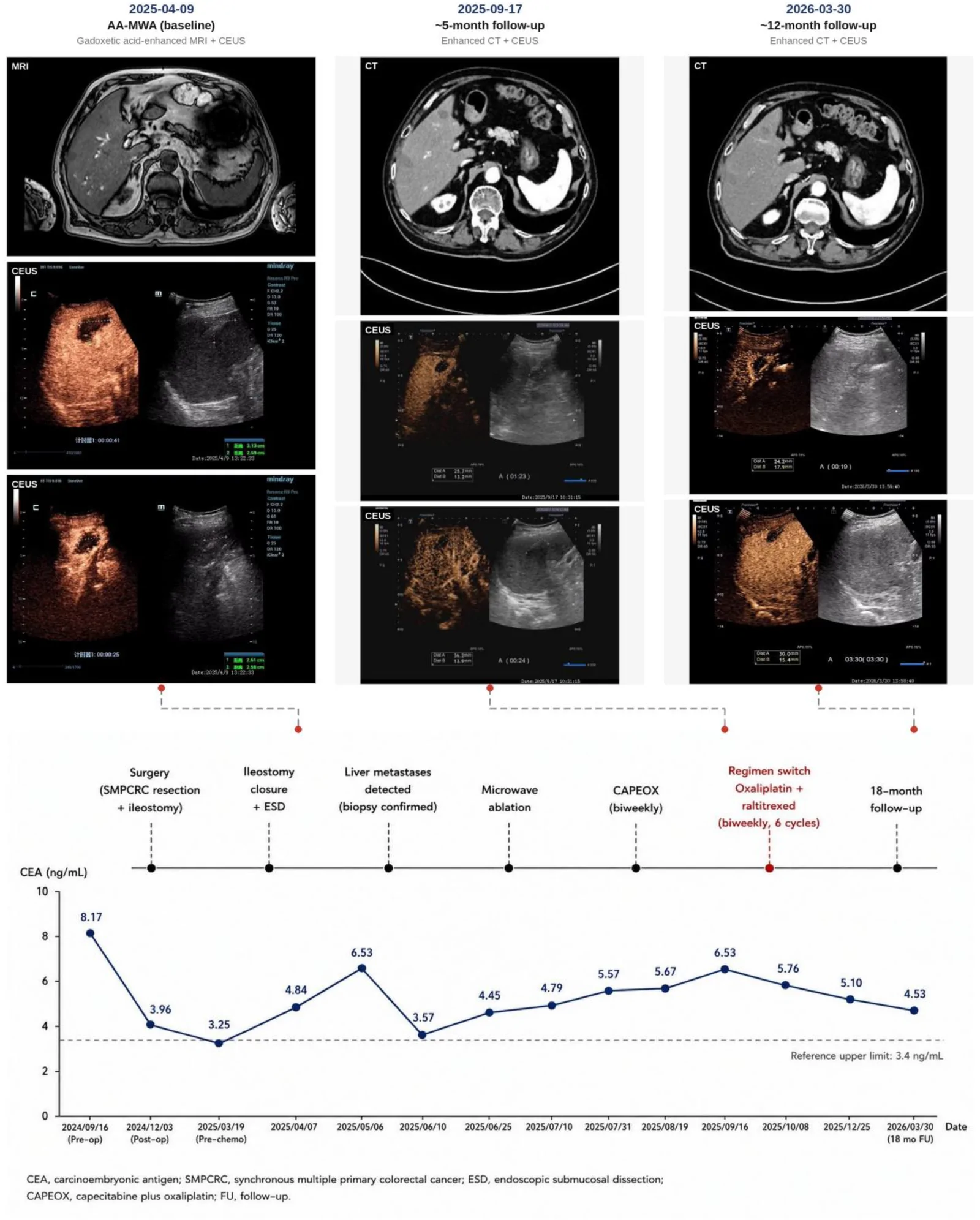
Dynamic changes of liver imaging and serum CEA level over the treatment and follow-up course. Top, liver imaging at three key follow-up time points: 2025-04-09 (intraoperative AA-MWA/baseline; gadoxetic acid-enhanced MRI and CEUS), 2025-09-17 (∼5-month follow-up; enhanced CT and CEUS), and 2026-03-30 (∼12-month follow-up; enhanced CT and CEUS); all time points indicate complete tumor ablation without new metastases. Bottom, dynamic changes of serum CEA level and key treatment events over 18 months of follow-up; the dashed horizontal line indicates the reference upper limit (3.4 ng/mL). Dashed connectors link each imaging time point to the corresponding date on the CEA curve. AA-MWA, artificial ascites-assisted percutaneous microwave ablation; MRI, magnetic resonance imaging; CT, computed tomography; CEUS, contrast-enhanced ultrasound.

The dynamic changes in serum CEA and the radiological follow-up of liver imaging during the 18-month follow-up period are shown in Figure 2. The preoperative CEA level was elevated (8.17 ng/mL) and decreased to normal after surgery; however, at the diagnosis of liver metastasis the CEA level was still within the reference range (3.25 ng/mL), indicating that the tumor marker failed to provide early warning of metastasis. During CAPEOX treatment, the CEA level instead rose to 6.53 ng/mL; after the regimen was adjusted to oxaliplatin plus raltitrexed, the CEA level decreased markedly within 5 weeks and remained at a low level with fluctuation during treatment, while imaging remained stable.

**Figure 3:**
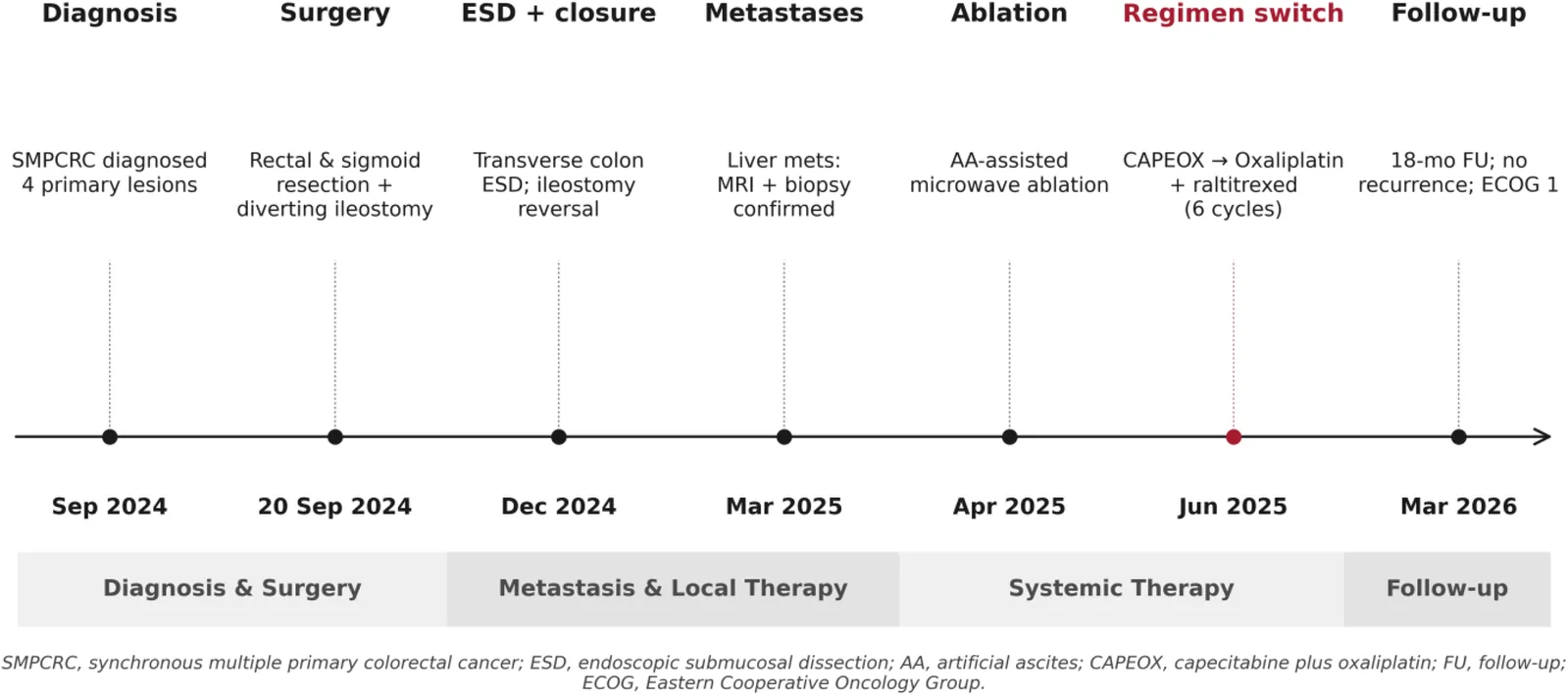
Timeline of the treatment course and key clinical events. Black dots indicate routine clinical events; the red dot highlights the chemotherapy regimen switch from CAPEOX to oxaliplatin plus raltitrexed prompted by CAPEOX-related grade 3 diarrhea. AA, artificial ascites; CAPEOX, capecitabine plus oxaliplatin; ECOG, Eastern Cooperative Oncology Group; ESD, endoscopic submucosal dissection; FU, follow-up; SMPCRC, synchronous multiple primary colorectal cancer.

CEUS at approximately one year after ablation showed heterogeneous areas of mixed echogenicity measuring 27 mm × 19 mm in the left hepatic lobe and 31 mm × 19 mm in the right hepatic lobe; no contrast agent filling was seen in the arterial, portal, or delayed phases, presenting as filling defect areas (24 mm × 18 mm and 30 mm × 15 mm), and no obvious abnormal washout area was seen on whole-liver scanning in the delayed phase, consistent with the sonographic appearance of complete tumor ablation, with no new metastasis. The dynamic changes in liver imaging at each follow-up time point and the concurrent serum CEA curve are shown in Figure 2. At the most recent follow-up, the patient's overall survival had reached 18 months, with stable imaging, good general condition, and an ECOG score of 1. The overall treatment course and key clinical milestones are shown in Figure 3.

## 3 Discussion

### 3.1 Molecular heterogeneity between multiple lesions and its implications for treatment decisions

The core finding of this case is the marked discordance in RAS status between the two primary carcinomas in the same patient: the primary rectal lesion carried mutation signals in both KRAS exon 4 and NRAS exon 2, whereas the sigmoid colon lesion was entirely RAS/RAF/PIK3CA wild-type. If the decision were based on either lesion alone, the indication for anti-EGFR therapy would yield diametrically opposite judgments (14, 15); in fact, current NCCN (6) and ESMO (5) guidelines do not provide explicit recommendations for multi-lesion molecular testing in SMPCRC, and some studies still use the molecular features of the index tumor as the basis for treatment (8). This strategy implicitly assumes that “a single sample can represent the molecular features of the entire disease.” This case directly demonstrates that this assumption may not hold in SMPCRC.

de Macedo et al. (7) found RAS discordance in 75% of eight SMPCRC cases, but the discordance in previous reports mostly manifested as a difference between a single KRAS locus and wild-type (7, 16). Malapelle et al. (17) highlighted the value of NGS-based genomic profiling in synchronous colonic carcinomas, showing that lesion-specific mutational signatures can help identify the primary lesion responsible for metastatic spread and refine tailored treatment. De Stefano et al. (18) reported apparent RAS discordance between a primary colon tumor and liver metastases that was resolved by tumor-cell enrichment and NGS, emphasizing the importance of specimen selection and analytical methodology. More recently, Lee et al. (19) used whole-exome sequencing in synchronous colorectal cancers and reported high intertumoral heterogeneity, recommending molecular analysis of all tumors when targeted therapy may be affected. Against this background, the primary rectal lesion simultaneously involved two genes, KRAS exon 4 and NRAS exon 2, with a more pronounced degree of heterogeneity — to our knowledge, this pattern of “one lesion involving variants in two RAS genes while another is entirely wild-type” is uncommon in SMPCRC. The co-occurrence of KRAS exon 4 and NRAS exon 2 signals within a single lesion may have several explanations that cannot be fully distinguished with the present data: a genuine concurrent mutation in two RAS genes; the presence of distinct subclonal populations within the same lesion, each carrying a different RAS variant; or, less likely, a technical artefact of the PCR fluorescent probe assay. Resolving these possibilities would require NGS with allele-frequency information, which could not be performed here because of the limited FFPE sample. It should be noted that this case used a PCR fluorescent probe method, which can detect mutation signals within an exon region but cannot precisely distinguish the specific loci within the region covered by the probe set (see Section 2.5); the specific amino acid changes still require confirmation by Sanger sequencing or NGS. This limitation does not affect the core conclusion that “the RAS status of the two primary lesions is clearly discordant.”

The molecular discordance between primary lesions supports a “multicentric origin” for this case (20), that is, the different tumors arise from independent clonal evolution rather than from the spread of a common ancestral cell. Of note, right- and left-sided colorectal cancers are known to differ in their molecular profiles, which provides a further conceptual basis for the inter-lesional heterogeneity observed across anatomically distinct primary tumors in this patient. In this case, the extensive polypoid lesions throughout the bowel and the evidence of the adenoma-carcinoma sequence (21) further fit the “field cancerization” mechanism (22, 23), in which a region of mucosa, under a common carcinogenic environment, gives rise to multiple independent pre-tumor regions, each subsequently undergoing parallel tumor evolution (24). The direct clinical implication of this mechanistic interpretation is that separate testing of multiple lesions is not merely an “additional safety net” but reflects the potential biological nature of SMPCRC.

In addition, at the diagnosis of liver metastasis in this case, the CEA level was still within the reference range, and the metastasis was detected by radiological examination rather than warned of by the tumor marker. Given the limited sensitivity of CEA for detecting recurrence (reported as approximately 50%–80%), a substantial proportion of CRC recurrences or metastases may occur without an elevation in CEA (12, 13), suggesting that in patients with molecular heterogeneity such as SMPCRC, structured radiological follow-up should not be relaxed because of a negative CEA result.

### 3.2 Multimodal comprehensive treatment in elderly patients

Another core feature of this case is that, in the complex scenario of “an elderly patient + four independent lesions + synchronous detection + secondary liver metastasis + intolerance to standard chemotherapy,” stable disease control was achieved for more than 18 months through a staged, multimodal integrated strategy — this integrated practice itself merits documentation as a feasible model for the treatment of complex SMPCRC. The integrated strategy included: synchronous management of three rectal/sigmoid primary lesions at the first operation to avoid the cumulative trauma of staged laparotomy (25); minimally invasive ESD rather than salvage surgery for the fourth lesion in the transverse colon detected shortly after surgery; AA-MWA rather than hepatectomy for bilobar liver metastases; and individualized adjustment of the systemic chemotherapy regimen according to tolerance.

When the basal margin of a transverse colon ESD specimen is positive, the standard approach is salvage radical surgery. However, in this case, after the multidisciplinary team assessed the patient's advanced age, overall tumor burden, and the risk of repeat laparotomy, close endoscopic follow-up was chosen instead of salvage surgery; biopsy of the original scar three months after ESD showed only inflammatory changes, and no evidence of local recurrence was found on subsequent follow-up. This result suggests that, in carefully selected elderly patients (in this case, a non-muscle-invasive resection with a positive margin, a limited overall tumor burden, and conditions allowing regular follow-up), the strategy of “ESD + scar biopsy + active surveillance” may be a feasible alternative to salvage surgery. The same rationale applies to the transanal local resection of the lesion 3 cm from the anal verge in this case — complete resection of the intramucosal carcinoma with close follow-up has achieved oncological control.

For elderly patients intolerant to standard chemotherapy, two aspects of the management in this case merit mention. First, AA-MWA was used to ablate the subcapsular hepatic lesion, effectively reducing the risk of thermal injury to the diaphragm and adjacent bowel and making it possible to treat a location that would otherwise be difficult to ablate safely (26, 27). Second, because of CAPEOX-related grade 3 diarrhea, the regimen was adjusted to oxaliplatin plus raltitrexed — raltitrexed, as a thymidylate synthase inhibitor, can be used in patients intolerant to fluoropyrimidines (28), and in this case the patient completed six subsequent cycles with improved tolerance. In retrospect, the absence of pretreatment DPYD assessment remains an important limitation, because genotype-guided dosing recommendations are available for patients with reduced DPD activity (29). Given the patient's advanced age, anti-EGFR, immunotherapy, and antiangiogenic treatment were not added in this case; instead, a comprehensive trade-off was made based on age, molecular features, and tolerance. This “subtractive” treatment rationale has reference value for similar complex elderly patients.

### 3.3 Limitations

This case has several limitations. Most importantly, the genetic testing in this case was based only on a PCR fluorescent probe method, which cannot precisely distinguish the specific amino acid changes among the loci covered by the probe set; because of the limited FFPE sample, supplementary Sanger or NGS confirmation could not be performed, so the “mutations” reported in this article should strictly be understood as “mutation-related signals.” In addition, the transverse colon lesion and the liver metastasis were not subjected to genetic testing; for the liver metastasis, only a small amount of biopsy tissue was available for morphology and immunohistochemistry (CK20, CK8/18, CDX2, and SATB2 positive; CK7 and Hepatocyte negative), and no resected metastatic specimen was available for additional molecular analysis. Germline genetic testing was not performed, so hereditary polyposis syndromes (such as attenuated FAP and MAP) cannot be completely excluded. In addition, TP53 was not included in the molecular panel used here: somatic TP53 status, which can be discordant between synchronous tumors, could therefore not be used to further clarify the clonal relationships among the lesions, and a germline TP53 alteration (Li-Fraumeni syndrome) could not be formally excluded, although the latter is clinically very unlikely given the advanced age at first presentation and the absence of a relevant personal or family history. Because only two of the four lesions were molecularly profiled, the clonal relationships among all lesions — and between the primary lesions and the liver metastasis, which may itself differ genetically from its lesion of origin (30) — cannot be definitively established, and the “multicentric origin” interpretation should therefore be regarded as the most plausible explanation rather than a proven one. DPYD testing was also not performed before capecitabine initiation; consequently, DPD deficiency cannot be excluded as a contributor to the grade 3 diarrhea. As a single case report, the conclusions of this article still require further validation by additional cases and prospective studies.

## 4 Conclusions

We report a 77-year-old man with synchronous multiple primary colorectal cancer presenting with four independent neoplastic lesions, marked discordance in RAS status between primary lesions, and liver metastasis detected by radiological evaluation despite a negative CEA, who achieved stable disease control for more than 18 months through staged multimodal integrated treatment. This case offers two core insights for clinical practice: (1) primary lesions in SMPCRC can harbor substantial molecular heterogeneity, and single-lesion testing may be insufficient to guide treatment decisions in complex scenarios — in this case, testing only the sigmoid colon lesion would have led to a judgment of the indication for anti-EGFR therapy opposite to the actual situation; and (2) for complex patients with “advanced age + multiple lesions + secondary liver metastasis + intolerance to standard chemotherapy,” a staged multimodal integrated strategy (synchronous surgery + ESD + AA-MWA + individualized chemotherapy adjustment) may serve as a feasible comprehensive option beyond salvage surgery or standard systemic therapy. These observations are based on a single case and still require further validation by additional cases and prospective studies.

## Data Availability

The original contributions presented in the study are included in the article/supplementary material, further inquiries can be directed to the corresponding authors.

## References

[1] Sung H Ferlay J Siegel RL Laversanne M Soerjomataram I Jemal A. Global cancer statistics 2020: GLOBOCAN estimates of incidence and mortality worldwide for 36 cancers in 185 countries. CA Cancer J Clin. (2021) 71(3):209–49. 10.3322/caac.21660 33538338

[2] Warren S Gates O. Multiple primary malignant tumors: a survey of the literature and a statistical study. Am J Cancer. (1932) 16:1358–414.

[3] Lam AK Chan SS Leung M. Synchronous colorectal cancer: clinical, pathological and molecular implications. World J Gastroenterol. (2014) 20(22):6815–20. 10.3748/wjg.v20.i22.6815 24944471 PMC4051920

[4] Mulder SA Kranse R Damhuis RA De Wilt JHW Ouwendijk RJT Kuipers EJ. Prevalence and prognosis of synchronous colorectal cancer: a Dutch population-based study. Cancer Epidemiol. (2011) 35(5):442–7. 10.1016/j.canep.2010.12.007 21470938

[5] Van Cutsem E Cervantes A Adam R Sobrero A Van Krieken JH Aderka D. ESMO consensus guidelines for the management of patients with metastatic colorectal cancer. Ann Oncol. (2016) 27(8):1386–422. 10.1093/annonc/mdw235 27380959

[6] National Comprehensive Cancer Network. NCCN clinical practice guidelines in oncology: colon cancer (version 5.2025); rectal cancer (version 4.2025). Available online at: https://www.nccn.org (Accessed May 15, 2026)

[7] de Macedo MP De Melo FM Da Silva Ribeiro J Lopes de Mello CA De Souza Begnami MDF Soares FA. RAS mutations vary between lesions in synchronous primary colorectal cancer: testing only one lesion is not sufficient to guide anti-EGFR treatment decisions. Oncoscience. (2015) 2(2):125–30. 10.18632/oncoscience.118 25859555 PMC4381705

[8] Simu P Jung I Banias L Kovacs Z Fulop ZZ Bara T. Synchronous colorectal cancer: improving accuracy of detection and analyzing molecular heterogeneity-the main keys for optimal approach. Diagnostics (Basel). (2021) 11(2):314. 10.3390/diagnostics11020314 33671994 PMC7919277

[9] Sorich MJ Wiese MD Rowland A Kichenadasse G McKinnon RA Karapetis CS. Extended RAS mutations and anti-EGFR monoclonal antibody survival benefit in metastatic colorectal cancer: a meta-analysis of randomized, controlled trials. Ann Oncol. (2015) 26(1):13–21. 10.1093/annonc/mdu378 25115304

[10] Zhang J Zheng J Yang Y Lu J Gao J Lu T. Molecular spectrum of KRAS, NRAS, BRAF and PIK3CA mutations in Chinese colorectal cancer patients: analysis of 1,110 cases. Sci Rep. (2015) 5:18678. 10.1038/srep18678 26691448 PMC4687048

[11] Zelli V Parisi A Patruno L Cannita K Ficorella C Luzi C. Concurrent RAS and RAS/BRAF V600E variants in colorectal cancer: more frequent than expected? A case report. Front Oncol. (2022) 12:863639. 10.3389/fonc.2022.863639 35463316 PMC9022079

[12] Sørensen CG Karlsson WK Pommergaard H-C Burcharth J Rosenberg J. The diagnostic accuracy of carcinoembryonic antigen to detect colorectal cancer recurrence - a systematic review. Int J Surg. (2016) 25:134–44. 10.1016/j.ijsu.2015.11.065 26700203

[13] Locker GY Hamilton S Harris J Jessup JM Kemeny N Macdonald JS. ASCO 2006 update of recommendations for the use of tumor markers in gastrointestinal cancer. J Clin Oncol. (2006) 24(33):5313–27. 10.1200/JCO.2006.08.2644 17060676

[14] De Roock W Jonker DJ Di Nicolantonio F Sartore-Bianchi A Tu D Siena S. Association of KRAS p.G13D mutation with outcome in patients with chemotherapy-refractory metastatic colorectal cancer treated with cetuximab. JAMA. (2010) 304(16):1812–20. 10.1001/jama.2010.1535 20978259

[15] Karapetis CS Khambata-Ford S Jonker DJ O'Callaghan CJ Tu D Tebbutt NC. K-ras mutations and benefit from cetuximab in advanced colorectal cancer. N Engl J Med. (2008) 359(17):1757–65. 10.1056/NEJMoa0804385 18946061

[16] Schirripa M Cremolini C Loupakis F Morvillo M Bergamo F Zoratto F. Role of NRAS mutations as prognostic and predictive markers in metastatic colorectal cancer. Int J Cancer. (2015) 136(1):83–90. 10.1002/ijc.28955 24806288

[17] Malapelle U De Stefano A Carlomagno C Bellevicine C Troncone G. Next-generation sequencing in the genomic profiling of synchronous colonic carcinomas: comment on Li et al. (2015). J Clin Pathol. (2015) 68(11):946–7. 10.1136/jclinpath-2015-203205 26139632

[18] De Stefano A Rosanova M Malapelle U Martini M De Falco S Attademo L. Discordance in RAS mutations between primary colon tumor and metastases: a real event or a matter of methodology? Int J Biol Markers. (2017) 32(4):e474–7. 10.5301/ijbm.5000273 28665451

[19] Lee HG Kim Y Kim M-J Kim YW Jun S-Y Kim D. Molecular mosaics: unveiling heterogeneity in synchronous colorectal cancers. Cancer Res Treat. (2026) 58(1):264–74. 10.4143/crt.2024.947 39973140 PMC12800958

[20] Eguchi K Yao T Konomoto T Hayashi K Fujishima M Tsuneyoshi M. Discordance of p53 mutations of synchronous colorectal carcinomas. Mod Pathol. (2000) 13(2):131–9. 10.1038/modpathol.3880024 10697269

[21] Fearon ER Vogelstein B. A genetic model for colorectal tumorigenesis. Cell. (1990) 61(5):759–67. 10.1016/0092-8674(90)90186-I 2188735

[22] Slaughter DP Southwick HW Smejkal W. Field cancerization in oral stratified squamous epithelium: clinical implications of multicentric origin. Cancer. (1953) 6(5):963–8. 10.1002/1097-0142(195309)6:5<963::AID-CNCR2820060515>3.0.CO;2-Q 13094644 10.1002/1097-0142(195309)6:5<963::aid-cncr2820060515>3.0.co;2-q

[23] Curtius K Wright NA Graham TA. An evolutionary perspective on field cancerization. Nat Rev Cancer. (2018) 18(1):19–32. 10.1038/nrc.2017.102 29217838

[24] Sottoriva A Kang H Ma Z Graham TA Salomon MP Zhao J. A big bang model of human colorectal tumor growth. Nat Genet. (2015) 47(3):209–16. 10.1038/ng.3214 25665006 PMC4575589

[25] Papamichael D Audisio RA Glimelius B De Gramont A Glynne-Jones R Haller D. Treatment of colorectal cancer in older patients: international society of geriatric oncology (SIOG) consensus recommendations 2013. Ann Oncol. (2015) 26(3):463–76. 10.1093/annonc/mdu253 25015334

[26] Zhang M Liang P Cheng ZG Yu XL Han ZY Yu J. Efficacy and safety of artificial ascites in assisting percutaneous microwave ablation of hepatic tumours adjacent to the gastrointestinal tract. Int J Hyperthermia. (2014) 30(2):134–41. 10.3109/02656736.2014.891765 24571176

[27] Song I Rhim H Lim HK Kim YS Choi D. Percutaneous radiofrequency ablation of hepatocellular carcinoma abutting the diaphragm and gastrointestinal tracts with the use of artificial ascites: safety and technical efficacy in 143 patients. Eur Radiol. (2009) 19(11):2630–40. 10.1007/s00330-009-1463-x 19557416

[28] Avallone A Gennaro ED Silvestro L Iaffaioli VR Budillon A. Targeting thymidylate synthase in colorectal cancer: critical re-evaluation and emerging therapeutic role of raltitrexed. Expert Opin Drug Saf. (2014) 13(1):113–29. 10.1517/14740338.2014.845167 24093908

[29] Amstutz U Henricks LM Offer SM Barbarino J Schellens JHM Swen JJ. Clinical pharmacogenetics implementation consortium (CPIC) guideline for dihydropyrimidine dehydrogenase genotype and fluoropyrimidine dosing: 2017 update. Clin Pharmacol Ther. (2018) 103(2):210–6. 10.1002/cpt.911 29152729 PMC5760397

[30] Vakiani E Janakiraman M Shen R Sinha R Zeng Z Shia J. Comparative genomic analysis of primary versus metastatic colorectal carcinomas. J Clin Oncol. (2012) 30(24):2956–62. 10.1200/JCO.2011.38.2994 22665543 PMC3417049

